# Editorial: Mental health in older adults with cognitive impairments and dementia: a multidisciplinary perspective

**DOI:** 10.3389/fpsyt.2023.1297903

**Published:** 2023-09-29

**Authors:** Dongdong Jiang, Xin Xu, Zuyun Liu, Qian Yang

**Affiliations:** ^1^Department of Geriatrics of the Fourth Affiliated Hospital, School of Public Health, Zhejiang University School of Medicine, Zhejiang University, Hangzhou, China; ^2^School of Public Health and the Second Affiliated Hospital of School of Medicine, Zhejiang University, Hangzhou, China; ^3^The Key Laboratory of Intelligent Preventive Medicine of Zhejiang Province, Department of Big Data in Health Science School of Public Health, Center for Clinical Big Data and Analytics Second Affiliated Hospital, Zhejiang University School of Medicine, Hangzhou, Zhejiang, China

**Keywords:** psychological and mental health, older adults, multidisciplinary, cognitive impairment and dementia, global health

## Why we initiate this Research Topic

Population aging has become an irreversible global trend. The number of people over 60 years old worldwide has surpassed 1.1 billion, with rising prevalence of cognitive impairment and dementia. Extensive literature has evidenced the high incidence rates and detrimental impacts of mental illnesses among older adults (1, 2). Older patients with cognitive decline or dementia coupled with psychiatric conditions are generally more susceptible to medical risks owing to the chronic and costly nature of such disorders. Moreover, public health emergencies, racism, climate change and other social or policy changes can also potentially affect overall treatment regimens, exacerbating existing mental health problems among older people with cognitive decline or dementia. Reports indicate that multidisciplinary approaches have become mainstream in this field, with interdisciplinary scholars examining geriatric mental health through an expanded lens. They account for not only the illnesses themselves, but also social impacts, neural mechanisms, genetic influences, and policy guidance to enhance standards of care holistically.

Therefore, this Research Topic applies a multidisciplinary perspective from medical and social domains (e.g., epidemiology, geriatrics, genomics, psychiatry, sociology, management) utilizing diverse methodologies and theories. It provides critical insight into mental health mechanisms, accessibility, utilization, strategies, social indicators, and outcomes for those with cognitive impairments and dementia. The aim for this Research Topic is to facilitate communication and disseminate the latest advancements across countries, cultures, and disciplines.

## Overview of the Research Topic

This Research Topic, “*Mental health in older adults with cognitive impairments and dementia: a multidisciplinary perspective*,” focuses on utilizing multidisciplinary approaches to address mental health issues in the elderly with potential or existing cognitive deficits and dementia. It currently comprises eight published articles—six original research, one review, and one brief research report. The contributors to this Research Topic, coming from diverse backgrounds and expertise, have certainly fulfilled the objectives through their investigations on mental health among older people. In order to make these many excellent contributions clearer to the readers, we divided these eight articles in this Research Topic into four sections: (1) different cultural contexts and minorities; (2) nursing-related practices in different regions in China; (3) dementia-related psychological and behavioral manifestations; (4) the role of social supports.

### Different cultural contexts and minorities

There are three articles which focusing on the mental health issues of older people with underlying or existing cognitive impairment and dementia in different cultural context and minority. Wang et al. conducted a review of 13 articles on filial piety culture and the wellbeing of family caregivers. Their findings indicate that reciprocal filial piety is beneficial for reducing the burden and pressure on children who care for parents with dementia. In a survey of 1,262 older individuals from Hakka families, Chang et al. discovered that reducing health-related behaviors among ethnic minority older adults can enhance their mental health. The last study (Aranda et al.) in this section found that a history of psychiatric and substance use disorders increases the risk of developing Alzheimer's disease (AD) and vascular dementia (VaD) in later life. The authors recommend that doctors and caregivers exercise caution when prescribing psychiatric medications to underrepresented older racial/ethnic minority groups.

### Nursing-related practices in different regions in China

Interestingly, there two articles concerning the same discipline (nursing) but using analytical methods from different disciplines. Sun et al. utilized management methods to evaluate and analyze the performance of the long-term care system for older individuals with disabilities and dementia in Zhejiang Province. Their findings indicate that the long-term care system in Zhejiang Province requires further improvement in terms of fairness, accessibility, and affordability. In contrast, a study conducted in Shandong Province by Chen et al. focused on caregivers for dementia patients and employed a socio-epidemiological approach. The study revealed that caregivers exhibited only moderate confidence and lacked a people-oriented nursing philosophy and methods.

### Dementia-related psychological and behavioral manifestations

Only one article in our Research Topic addresses dementia-related psychological and behavioral manifestations. Yang et al. utilized cohort data from China to assess the influence of baseline cognitive performance, current cognitive function, and cognitive decline on subsequent depression symptoms in older adults. Their findings revealed that individuals who experienced significant cognitive decline were more likely to develop depression.

### The role of social supports

Numerous studies have consistently demonstrated the effectiveness of social support in preventing mental disorders (3, 4). In this Research Topic, Zhu et al. identified key factors for preventing mild cognitive impairment (MCI) among older individuals, which include providing appropriate social activities and creating a supportive environment. Additionally, Gu et al. highlighted the differences in social connectivity between urban and rural older adults, emphasizing the need for targeted social support strategies from both family and society.

## Recommendation and conclusion

Mental health in older adults with underlying and existing cognitive impairment or dementia represents a chronic and systemic issue. Hence, adopting multidisciplinary lenses and approaches is an efficacious way to elevate mental wellbeing in elderly individuals with cognitive deficits, especially dementia. It necessitates attention and substantial support across all societal sectors.

We express our gratitude to the authors for their valuable and insightful contributions to this Research Topic. Furthermore, we hope that this Research Topic will contribute to the existing literature on mental health issues in older individuals with cognitive impairment and dementia, fostering interdisciplinary thinking and the utilization of diverse methodologies.

## Author contributions

DJ: Conceptualization, Writing—original draft, Writing—review and editing. XX: Supervision, Validation, Writing—review and editing. ZL: Supervision, Validation, Writing—review and editing. QY: Project administration, Resources, Supervision, Validation, Writing—original draft, Writing—review and editing, Conceptualization.
